# Learning health care systems: Highly needed but challenging

**DOI:** 10.1002/lrh2.10211

**Published:** 2020-01-13

**Authors:** Roel H.P. Wouters, Rieke van der Graaf, Emile E. Voest, Annelien L. Bredenoord

**Affiliations:** ^1^ Department of Medical Humanities, Julius Center for Health Sciences and Primary Care University Medical Center Utrecht/Utrecht University Utrecht The Netherlands; ^2^ Department of Medical Oncology Netherlands Cancer Institute Amsterdam The Netherlands

**Keywords:** health policy, informed consent, learning health care system, learning health system, research ethics

## Abstract

**Background:**

Learning health care systems (LHSs) have the potential to transform health care. However, this transformation process faces significant challenges.

**Materials and methods:**

Based on proposals and early examples of LHSs in the literature and conceptual analysis of the LHS mission, we provide four models with distinct organizational and ethical implications that may facilitate the transformation.

**Results:**

An LHS could be developed in the following ways: by taking away practical impediments that prevent patients and professionals from engaging in scientific research (model 1: optimization LHS); by routinely analyzing observational data from electronic health records and other sources (model 2: comprehensive data LHS); by making clinical decisions based on the outcomes of the aforementioned data analyses and directly evaluating the outcomes in order to continuously improve decision‐making (model 3: real‐time LHS); or by embedding clinical trials into routine care delivery (model 4: full LHS).

**Conclusions:**

Each model has different ethical implications for consent and oversight. Also, the four‐model approach shows that reorganizing a health care center into an LHS is not an all‐or‐nothing decision. Rather, it is a choice from a menu of possibilities. Instead of discussing the advantages and disadvantages of the LHS menu in its entirety, the medical community should focus on the designs and ethical aspects of each of the separate options.

## 1 INTRODUCTION

For decades, research and clinical care have been viewed, both legally and practically, as separate entities within health care institutions. With the current rapid innovation in health care, many people characterize the strict distinction between care and research as troublesome. The dichotomy has been criticized for hampering scientific and medical progress while falling short of providing meaningful protections for research participants and patients in return.^1^, ^2^ In 2007, the US Institute of Medicine called upon health care leaders to transform their practices into learning health care systems (LHSs) so that research and care will be integrated and the health care activities will be continuously studied, learned from, and improved.^3^ This development is promising for precision medicine, as therapies can more accurately be tailored to specific patient characteristics if data from routine care would more systematically be used to generate clinical evidence.^4^ The American Society of Clinical Oncology (ASCO) has embraced the goal to derive real‐world evidence from daily practice and has subsequently launched its own LHS: CancerLinQ.^5^ The goals of the LHS have also been embraced by the American Heart Association.^6^ In Europe, several national and international collaborations have been established to follow suit, for example, the EUROCAT initiative that is committed to building a cross‐border LHS for radiation oncology.^7^, ^8^

Thus far, few attempts to transform health care practices into an LHS have been completed.^8^ Although progress has been made and some examples of actual implementation have emerged, the preponderance of the current body of literature consists of conceptual ideas and strategies. Various barriers have been reported, including cultural reluctance to change among health care professionals, limited quality and interoperability of clinical data stored in electronic health records (EHRs), and ethical and/or legal constraints on amending informed consent procedures.^9^, ^10^ However, what has largely been overlooked in the discussion is that the LHS‐concept itself is not uniform. An analysis of the learning health care mission, proposals to achieve that mission, and early examples of actual implementation shows that the LHS can be differentiated into different models. In this paper, we propose a conceptual framework that aids the development of successful implementation policies by categorizing LHSs into four models with distinct methodological and ethical features, particularly attributes that bear on the distinction between clinical care and research.

## 2 METHODS

A narrative review of the relevant literature was performed. We compared the literature on methodological, organizational, and ethical aspects of LHSs. This review devoted particular attention to how these systems combine the pursuit of scientific medical evidence and the provision of patient care. Next, a conceptual analysis of the LHS scholarship was used to categorize the variety of proposals into four different models. We constructed a framework that, on the one hand, illuminates the diversity and heterogeneity of (proposed) learning health care systems and, on the other hand, clarifies how each set of proposals correlates with different ethical issues. The taxonomy was improved upon through an iterative process, in which continued discussion among the authors led to revised categorizations that were subsequently evaluated and refined by determining how well various learning health care systems and their specific ethical challenges fit into the models. Finally, we arrived at a four‐model approach that most adequately grasps the diversity of learning health care systems within each model as well as the ethical issues raised by these systems.

### 2.1 Optimization learning health care system (model 1)

Integrating care and research activities often evokes visions of legal reform and lengthy administrative procedures. Aligning care and research does not, however, always necessitate removal of structural barriers between care and research. For example, innovative study designs have been developed to increase the number of patients participating in clinical research and to yield research results that better represent daily clinical care. One such approach is Trial within Cohorts, an innovative design in which a research cohort of patients functions as a control arm for multiple intervention studies and as a platform for recruitment in intervention arms of those studies.^11^ Trials within Cohorts (also referred to as cohort multiple randomized controlled trials) have been used to study interventions for cardiovascular diseases as well as multiple types of malignancies.^12^, ^13^, ^14^ In addition, similar protocols have been adopted by researchers working in diverse settings, including primary care and specialized hospitals, to study a broad range of medical conditions, from cystic fibrosis to HIV.^15^ Although not all of these applications are labelled explicitly as an LHS, they do encompass strategies that contribute to a health care system in which “the process of generating and applying the best evidence will be natural and seamless components of the process of care itself.”^3^ Implementing innovative study designs may promote scientific research in a way that is more congruous with daily care delivery, while key ethical and legal distinctions between care and research (eg, informed consent, independent ethics review) continue to exist.

We call these efforts “optimization LHSs” because conditions within care and research structures are optimized to create an environment that encourages patients to participate in research and professionals to translate scientific insights into clinical practice. Crucially, patients in this type of LHS are included as participants in a study, explicitly crossing the Rubicon from care to research. Other examples of the optimization LHS, besides the aforementioned innovative designs, are specifically geared toward shifting the culture in a health care practice. Practices may become more innovative, for instance, by employing health care professionals who are more science‐minded, dedicating more staff to recruitment of patients in clinical trials, or promoting collaboration between clinicians and researchers.^8^ Comprehensive cancer centers, such as the Netherlands Cancer Institute, have shown to improve patient outcomes by creating such an innovation‐minded organization.^16^ Optimization can also be achieved by streamlining payment schemes in research and clinical care. Precision medicine increasingly enables identification of relatively small subgroups of patients who may benefit from treatment with off‐label medication, preferably in the setting of a clinical trial. However, both testing that is required for identification (e.g., genomic sequencing) and expenses for off‐label medication are typically not reimbursed by insurance companies or public payers.^17^, ^18^ Recently, personalized reimbursement schemes have been developed in which off‐label drugs are initially funded by research institutions and pharmaceutical companies, but eventually by insurance agencies in case clinical effects evidently occur.^19^ Such financial innovations bridge the gap between care and research, while they do not require legal or ethical reform.

### 2.2 Comprehensive data learning health care system (model 2)

**Figure 1:**
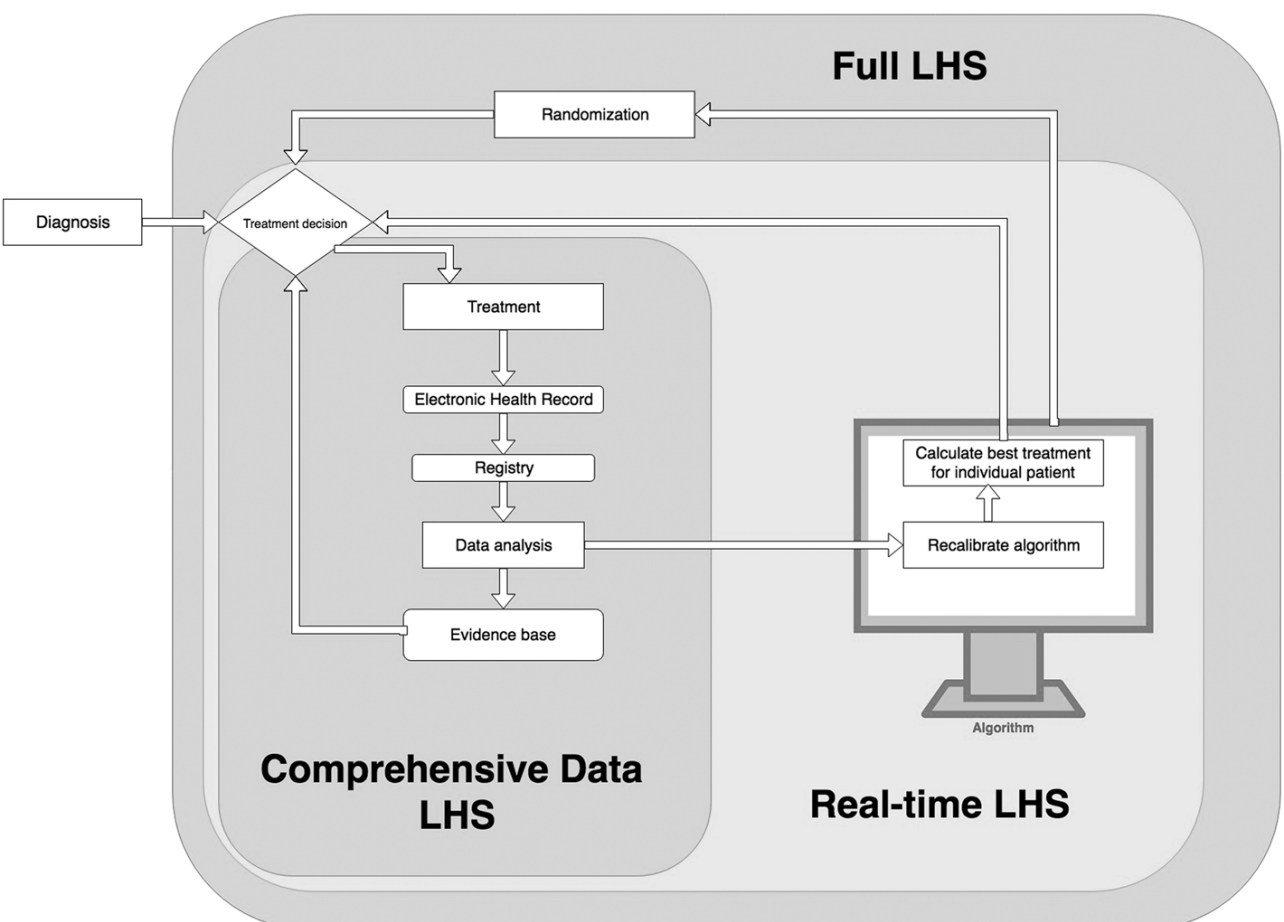
A schematic overview of comprehensive data, real‐time and full LHSs

Nowadays, care delivery produces massive amounts of data that are being collected and stored in EHRs, administrative databases, and quality management systems. One particular type of LHS unlocks the scientific potential of routine care data by streamlining the way in which these data are recorded, processed, and made available for research. The envisioned result is a continuous stream of systematically captured clinical data that can subsequently be analyzed to test and generate a wide variety of research hypotheses (Figure 1).^4^, ^20^ Recently, EHR‐based research initiatives have been developed in several countries to link data on baseline patient characteristics to biological parameters (such as biomarkers or genome profiles) and outcomes.^21^ For example, pediatric hospitals in the United States have joined forces to bring together data from local EHRs into a centralized database. Similar initiatives have been developed in several European countries, such as the NHS Digital project, which is committed to transforming the United Kingdom's National Health Service into a nationwide LHS by collecting and linking EHR data from both primary and secondary care practices.^22^, ^23^ We call such efforts “comprehensive data LHSs” because these LHSs generate evidence by routinely collecting and processing vast quantities of clinical data. From a methodological perspective, comprehensive data LHSs resemble longitudinal cohort studies, which are also observational and prospective in nature. However, traditional cohort studies are not embedded into the dataflow of routine care delivery but rather depend on data collection among patients explicitly included into a study cohort (under a specific research protocol). Furthermore, comprehensive data LHSs are set apart from retrospective medical record research because comprehensive data LHSs systematically collect, curate, and store data to facilitate research. Creating comprehensive LHSs increases integration of care and research, but research involvement remains nonexperimental in the sense that the care received by individual patients is not directly affected by the research‐oriented dataflow in the background. At the same time, the fact that patients become routinely involved in research through their data necessitates careful scrutiny of voluntariness and trust.

### 2.3 Real‐time learning health care system (model 3)

Establishing a comprehensive data LHS is often presented as the first step toward creating an LHS that provides real‐time feedback of data analysis at the point of care.^21^, ^24^ Real‐time learning health care builds on previous advances in the field of clinical decision‐support systems, such as applications that detect medication errors and warn physicians about potential drug interactions.^25^ Real‐time LHSs not only aim to provide a helpful tool for clinicians but also want to use digital feedback systems for the pursuit of generalizable knowledge. These ambitions may extend to virtually every corner of medicine but are particularly well articulated in oncology. ASCO's CancerLinQ explicitly envisions that data generated through clinical care are used not merely for observational research but also as input for rapid‐learning algorithms that guide clinical decision‐making for individual patients. Treatment decisions calculated by such algorithms are constantly changing, as the outcomes of each previously calculated treatment decision are fed back into a database and are used to improve the constantly recalibrating algorithm.^26^ Similar strategies are already being implemented by the US Department of Veterans Affairs (VA). In the VA Precision Oncology Program, lung cancer patients for whom tumor boards have no single recommendation for treatment pass through an algorithm‐aided process. In this process, the optimal treatment option is determined by comparing (among other factors) the patient's biomarker profiles with data from previous patients that are already in the database.^27^, ^28^ Currently, these and other initiatives are still fairly limited in scope; that is, they are being tested for specific diseases and patient populations. Moreover, they are conducted under a study protocol and not (yet) implemented in routine care workflows.^29^ Nevertheless, if proven successful, they may serve as a model for subsequent national and international real‐time LHSs.

The distinctive feature of a “real‐time LHS” is the combination of clinical decision‐support algorithms with routine clinical data collection. Combining these elements in a real‐time LHS allows professionals to translate novel insights derived from the treatment of previous patients directly into patient management, which in turn yields data that can be used to evaluate and fine‐tune clinical decisions in the future.^26^ This LHS model disrupts the traditional distinction between care and research because, in a real‐time LHS, patients receive care according to newly generated hypotheses that are subsequently tested and refined for the benefit of future patients.

### 2.4 Full learning health care system (model 4)

Finally,iIt has been proposed that typical trial methodologies, such as randomization, should be embedded in clinical care. For instance, comparative effectiveness trials may be integrated in routine care by randomizing patients between two or more standard of care interventions whose relative merits are unknown. By embedding such trials in EHRs, an LHS can recruit or even include all patients eligible for participation in the study. An early example of this type of LHS is the randomized registry trial, in which recruitment, randomization, and data collection were conducted using an existing population registry (initially set up for quality control).^30^ In this registry study, oral consent was obtained before patients were randomized between two interventions in a trial. However, others have suggested that consent can sometimes be omitted for comparative effectiveness studies if the interventions are considered standard of care.^1^ Some LHS proponents advocate a similar approach, pointing out that randomization can be integrated into self‐learning algorithms, randomly assigning patients to the highest‐ranking treatment decisions as calculated by the algorithm.^31^, ^32^

We think that such proposals are best described as a “full LHS” because they entail that patients who would normally be treated in a clinical care setting will be treated in a setting that is explicitly designed as a scientific study. In this scenario, research and care would be fully integrated. Whereas comprehensive data and real‐time LHSs produce generalizable knowledge as a by‐product of how care is organized, clinical care in a full LHS is remodeled on research principles to the extent that the care delivery process becomes a prospective interventional trial.

## 3 POLICY IMPLICATIONS FOR TRANSFORMATION TOWARD AN LHS

Despite the enthusiasm for the conceptual idea and early examples of successful LHSs, the LHS concept struggles with achieving synergy between care and research as was envisioned by the Institute of Medicine over a decade ago. One possible explanation for this gap between literature and practice is that the LHS policy proposals in theliterature are very heterogeneous. The LHS has initially been presented as a comprehensive reconceptualization and rearrangement of scientific inquiry vis‐à‐vis care.^3^ This set off a debate on very fundamental issues regarding the care‐research dichotomy. For example, it has been debated as to whether patients should be expected to be part of scientific research, whether randomized comparative effectiveness research may be embedded in clinical care, and whether patients in clinical care can justifiably be exposed to risks for the sake of acquiring generalizable knowledge.^2^, ^10^, ^33^ These intricacies may deter policy makers from pushing their organizations toward an LHS. However, at present, many LHS strategies that have been outlined do not propose such a far‐reaching dissolvement of care and research and therefore avoid many ethical and legal implications of an extensive rearrangement.

The models described above may provide health care practices with a more accessible and gradual approach to implementing one of the alternatives on the LHS spectrum. Health care practices committed to building an LHS are not required to go “all‐in” by building a full LHS. Many LHS goals can be achieved through stepwise transformation into one of the other models. Other LHS taxonomies, such as the Heimdall framework, have also pointed out that there is a diversity of options to establish an LHS and have contributed to a better understanding of how different LHS goals can be accomplished.^34^ The current framework adds to this previous work by explicitly linking subsets of LHSs to specific ethical challenges, thus helping policy‐makers to strike a balance between pursuing technical possibilities and avoiding ethical controversies. Likewise, our framework is conducive to identifying which LHSs require comprehensive ethics oversight reform and which may be initiated pending the outcomes of longer‐term reform efforts. For example, the goal of producing a culture shift toward a health care organization where scientific evidence gathering is a joint responsibility of all professionals can be realized through an optimization LHS.^3^ Furthermore, a comprehensive data LHS can be used to gather evidence on the real‐life effectiveness of therapeutics. Within large amounts of routinely assembled clinical (EHR) data, correlations may be detected that previously remained unnoticed in clinical trials because sample sizes were too small or study populations too homogeneous (for example, by excluding elderly patients with comorbidities).^4^ In fields of health care where genomics‐guided therapies are rapidly becoming the norm, such as in many areas of oncology, real‐time LHSs could be particularly useful. Especially in cases where no effective treatment options are available , matching patients to therapeutics on the basis of large quantities of (patient, biomarker, and clinical outcome) data, and subsequently evaluating the effects of this matching process, may enable a great leap forward in understanding diseases and improving prospects for patients.^24^, ^25^, ^26^

In addition to determining which model fits an organization's context and ambitions, health care leaders must consider whether a preferred LHS can be established in an ethically justified manner and whether it will be acceptable to the population. By utilizing day‐to‐day care delivery for the acquisition of scientific evidence, LHSs renegotiate the terms on which scientific research has typically been morally justified. Traditionally, exposing patients to risks for the purpose of scientific inquiry is only acceptable under strict ethical requirements, including informed consent and review by a research ethics committee.^1^, ^10^, ^33^ Informed consent has therefore been at the forefront of the LHS debate. Many argue that having to seek explicit informed consent from every patient at each step of the research process may introduce logistical challenges that are at odds with the LHS goal to seamlessly embed research into care.^3^, ^9^ Amendments to conventional informed consent norms can be necessary to produce knowledge that adequately reflects real‐life clinical conditions and to prevent the generation of this knowledge from impeding clinical care. Debating such amendments, one should account for the fact that different LHS models pose different types and levels of risk to patients. Privacy risks, such as confidentiality breaches and the risk that data will be used for research purposes that are not in conformity with the patient's personal values, are the primary source of risk in a comprehensive data LHS, compared with clinical care in a non‐LHS practice. Real‐time and full LHSs face not only privacy risks but also physical risks because the prescribed interventions can have harmful side effects or may eventually prove less efficacious than alternative treatment options.^35^

An important ethical reason to consider the transformation of a health care practice into an LHS is that the care‐research distinction offers limited guidance for determining the adequate level of ethics regulations and oversight.^36^ Quality improvement studies, for example, are often judged to be situated in a gray zone that cannot be classified as either care or research proper. Patients who encounter risks as a result of participating in such studies forgo the extensive protections offered to participants in human subjects research.^37^, ^38^ The same holds true for risk encountered by patients as a result of medical training.^2^ By contrast, many activities that are currently labeled as research do not pose more risk than ordinary care but are nevertheless subjected to stringent ethics oversight. For these reasons, the care‐research distinction has been criticized as vague and morally arbitrary.^1^, ^2^, ^36^ We agree that the development of an LHS could contribute to resolving the confusion surrounding the blurriness of the care‐research distinction, yet reframing the entire gray zone between those categories as an LHS does not in itself offer much guidance. The four‐model approach provides a framework to match appropriate checks and balances to different operationalizations of the LHS.

## 4 MOVING FORWARD

Although the transformation of health care into LHSs is still in an early stage, several examples of LHS models have emerged. Our four‐model approach and policy recommendations provide guidance as to how current practices can be reorganized into the desired LHS. In a rapidly changing world, LHSs may provide a solution to reshape and improve our health care.

## CONFLICT OF INTEREST

The authors declare no conflicts of interest.
